# Preparation of magnetic biochar functionalized by polyvinyl imidazole and palladium nanoparticles for the catalysis of nitroarenes hydrogenation and Sonogashira reaction

**DOI:** 10.1038/s41598-023-44292-3

**Published:** 2023-10-13

**Authors:** Pourya Mohammadi, Majid M. Heravi, Leila Mohammadi, Asma Saljooqi

**Affiliations:** 1 Department of Chemistry, Faculty of Physics and Chemistry, Alzahra University, PO. Box 1993891176, Vanak, Tehran, Iran; 2 Department of Chemistry, Shahid Bahonar University of Kerman, Kerman, Iran

**Keywords:** Environmental chemistry, Catalysis

## Abstract

Catalysts are essential materials in biotechnology, medicine, industry, and chemistry. On the other hand, recycling and using waste materials is important in economic efficiency and green chemistry. Thus, biochar was prepared from the stem and roots of the *Spear Thistle* to recover waste. After magnetizing the biochar, its surface was modified with polyvinyl imidazole. Finally, this modified biochar was decorated with Pd nanoparticles and used as a selective and recyclable nanocatalyst in the hydrogenation of nitroarenes and the Sonogashira reaction. The structure of this organic–inorganic nanocatalyst has been characterized by FESEM-EDS, XRD, FT-IR, TEM, and VSM techniques. In the hydrogenation reaction with the amount of 30 mg of nanocatalyst, the temperature of 50 °C in the water solvent, the reaction efficiency reached 99% for 30 min. In addition, under optimal conditions for the Sonogashira reaction: 1.0 mmol iodobenzene, 1.2 mmol phenylacetylene, 20 mg MBC-PVIm/Pd, 2 mmol K_2_CO_3_ in H_2_O at 50 C for 15 min, the reaction efficiency reached 95%. The recyclability of magnetic nanocatalysts was investigated and recognized this nanocatalyst can be used several times without notable loss of its activity.

## Introduction

The synthesis of aniline derivatives has drawn much attention due to its essential precursors for pesticides, construction pharmaceuticals, pigment dyes, agrochemicals, and fine chemicals^1^. Traditional synthetic methods for these compounds include transition metal-catalyzed, cross-coupling reactions of ammonia with aromatic boronic acids^2^, 2-phenol derivatives^3^, and aryl halides^4^ or reduction of the corresponding nitroarenes^5^. New methods to catalytic nitroarenes reduction are more efficient in terms of atoms and compatible with large-scale processes. In recent years, more environmentally-friendly catalytic protocols have been developed to replace the Bechamp process, which involved Fe/HCl as a reducing agent^6^. It has been reported that high-pressure hydrogenation of nitroarenes has been performed using a range of heterogeneous catalysts^7^. The heterogeneous commercial catalysts based on Ni, Au, Pd, or Pt can be used for this conversion, but they are not helpful for substituted nitroarenes because of their poor selectivity.

Yang et al.^8^ efficiently utilized primary unactivated alkyl iodides and bromides in the Sonogashira reaction for the first time using a palladium-carbene catalyst. The coupling of alkynes with organic halides, known as the Sonogashira reaction, is an effective approach for synthesizing various substituted alkynes, versatile synthetic intermediates, and essential biologically active compounds^9^. One of the challenges related to the Sonogashira reaction is when "unreactive" aryl chlorides are used as coupling partners due to the increased availability and lower cost of aryl chlorides compared to aryl bromides and iodides^8^. Metal-based cross-coupling reactions demonstrate very versatile and powerful processes in organic synthesis^10^. Reaction resulting in the bond formation of C(sp)-C(sp2) is usually essential in a broad range of organic methods^11^. The Sonogashira reaction, including vinyl or aryl halides coupling with terminal alkynes, has appeared as a choice^12^.

Palladium (Pd) can catalyze several chemical reactions, including hydrogenation^13,14^, oxidation^15,16^, and carbon–carbon coupling^17,18^. Pharmaceutical syntheses currently employ homogeneous palladium catalysts, but separating them from the products is usually difficult. Consequently, because of the very important waste produced in the pharmaceutical industry i.e., the consequential metal contamination of the products becomes a critical problem. It is possible to use heterogeneous catalysts instead of homogeneous catalysts, which can prepare some advantages, such as increased stability, low cost, simple separation from the products, and recyclability^19^. As compared with homogeneous catalysts, heterogeneous catalysts may have relatively lower selectivity and activity than homogeneous catalysts. Pd nanoparticles can be immobilized on different supports such as magnetic nanoparticles, inorganic oxides, polymers, or biochar nanoparticles^20^ to improve metal dispersions' stability. As a result of the application of other pathways from those traditionally accepted, these catalytic systems often exhibit novel properties.

A carbon-rich, porous solid is called biochar when it is formed when biomass decomposes in the absence of oxygen at moderate temperatures (350–700 °C)^21^. Technically, biochar is derived from biomass pyrolyzed at relatively low temperatures with a limited amount of oxygen^22^. One of the oldest industrial technologies developed by mankind is the production of charcoal, which reflects this process^23^. Biochar is a particular type of biocarbon in terms of its use as a chemical feed. It is described as a wide variety of carbonaceous materials emanating from all biological sources, such as microbial, plant, and animal. According to this definition, biochar is distinguished from hydrochar, which refers to the carbonization of organic compounds derived from biomass, under relatively low temperatures (130–250 °C) and high pressures (0.3–4.0 MPa)^24^. Amorphous carbons such as carbon black and activated carbon are also distinguished from biochars. Both biochar and activated carbon are amorphous carbons with abundant porosity, so structurally, there is no fundamental difference between them. Even biomass-derived activated carbon can be considered a special activated biochar. However, biochar unlike activated carbon generally has abundant surface functional groups such as OH, COOH, C=O, and C–O, which are modifiable and serve as a substrate for the preparation of different functionalized carbon materials.

Polymers such as polyvinyl alcohol and polyvinyl imidazole are utilized in non-covalent interaction and trapping because it has amino and hydroxyl groups, which readily connect with other compounds^25^. Ren and coworkers developed an efficient sewage sludge-derived biochar as a catalyst via an easy one-step pyrolysis process and used it to hydrogenate nitrophenols^26^. Gu et al. synthesized a graphene/biochar-supported nanoscale zero-valent-iron. This catalyst was prepared using rice husk and graphite as the raw materials, reducing the cost, and applied to remove nitrobenzene compounds from an aqueous solution^27^. Wei and coworkers^28^ applied nanoscale zero-valent iron in the removal of nitrobenzene from polluted wastewater or groundwater limited due to its absence of stability, simple aggregation, and iron leaching. Ahmad et al.^29^ synthesized temperature-dependent Fe1-N1-BC1 composites with high efficiency and stability from available precursors for removing heavy metals and organic pollutants from water.

Ferlin and coworkers^30^ report on the development of a waste minimization/valorization methodology used to the representative benchmark Sonogashira reaction conducted in a continuous-flow reactor, featuring a continuous-flow downstream membrane organic/aqueous separator to recover medium and products with minimal waste. Kempasiddaiah et al.^31^ reported the synthesis of low-cost mesoporous biomass carbon from waste radish leaves and was employed as support in preparing a palladium-based (BC-TPEA@Pd) heterogeneous catalyst.

In heterogeneous catalysis, ferrites with the general formula MIIFeIII2O4 (M=Fe, Co, and Ni) are well-known, widespread materials that are magnetically and structurally stable^32^. However, some groundbreaking work has been done with Fe3O4 nanoparticles in organic synthesis for the coupling of aldehydes, alkynes, and amines^33^ and for the synthesis of xanthene derivatives^34^. Typically, the materials are used as catalyst carriers in the form of nanoparticles rather than as the catalytic entity themselves^35–37^. Catalysts are used both in academia and in the industry for their stability, high surface areas, and ease of recovery. All factors cannot be established in many of the reported supports. The magnetization of catalysts can overcome these drawbacks since they can be readily isolated from reaction media with an external magnet and have a great surface area. Because magnetic biochar nanoparticles have the advantages of both biochar (such as stability, high surface area, and availability) and magnetic nanoparticles (such as the ability to separate by an external magnet), magnetic biochar nanoparticles are suitable to support magnetically recoverable catalysts.

In this work, we prepared a magnetic biochar (MBC) modified with polyvinyl imidazole and decorated with Pd nanoparticles (MBC-PVIm/Pd). As a catalyst for the Sonogashira reaction and hydrogenation of nitroarenes, its catalytic performance, reusability, and stability were evaluated. One of the essential advantages of this method is that it produces high yields and makes it easy to recover the catalyst.

## Experimental

### Chemicals and materials

All materials and reagents were prepared from Merck and Sigma-Aldrich. The leaves were bought from a local shop in Tehran. This work has been done with a plant that can be found in abundance in local shops and is not endangered or wild. Legislation, guidelines, and institution guidelines are followed in this study.

X-ray diffraction (XRD) patterns were carried out using a PW 1800 X-ray analyzer (with a range of 2θ = 10–80 degrees on 40 kV current and 40 mA with Cu Kα radiation. A vibrating-sample magnetometer (VSM) model was recorded with Meghnatis Kavir Kashan Co., Iran. A field-emission scanning electron microscope (FESEM, Tescan Mira3, Czech Republic) was used for studies. The melting point was measured by Electro-Thermal 9200 device. Images of transmission electron microscopy (TEM) were taken with a CM30, Philips, Germany operating at 300 kV.

### Synthesis of MBC-PVIm/Pd nanocatalyst

The nanocatalyst used in this work has been synthesized in several steps as follows:

- *Synthesis of biochar (BC)* To remove the impurities of the *Spear Thistle*, we first washed its stem and roots several times with deionized water. A particle size range of 0.9–2 mm was achieved by crushing the stems and roots of the *Spear Thistle*. Then, the prepared samples in 70 mL of deionized water were transferred into a Teflon autoclave reactor and heated at 185 °C for 24 h. The obtained product was separated by centrifugation, washed several times with water, and dried at room temperature.
- *Synthesis of biochar/Fe*_*3*_*O*_*4*_* (MBC)* 0.3 g of BC was dispersed in 120 mL of deionized water for 30 min. Then, 0.5 g FeCl_2_·4H_2_O, and 1.37 g FeCl_3_·6H_2_O were added to the above mixture at 60 °C for 6 h. After this time, 11 mL of NH_3_ solution was added to it and stirred for 1 h at the same temperature. Eventually, the magnetic biochar was separated by a magnet, washed several times with water, and dried at ambient temperature.
- *Synthesis of MBC-PVIm* First, 0.6 g of biochar/Fe_3_O_4_ was stirred in 20 mL of ethanol for 1 h. Then 10 mmol of 1-vinyl imidazole was dissolved in 3 mL of EtOH, added dropwise to MBC, and stirred for 2 h. Next, 0.06 g of potassium persulphate as an initiator was dissolved in 3 mL of deionized water and added dropwise to the above mixture. This mixture was stirred at 70 °C under a nitrogen atmosphere. Finally, the product was separated with a magnet, rinsed several times with water, and dried at ambient temperature.
- *Synthesis of MBC-PVIm/Pd nanocatalyst* First, 50 mg MBC-PVIm nanocomposite was stirred in 50 mL of acetonitrile for 30 min. Separately, 3.0 mg PdCl_2_ was dissolved in 70 mL of acetonitrile until obtained a clear yellow solution, then this solution was added to the first mixture and stirred at 60 °C for 24 h. 1.0 mL of hydrazine hydrate (80%) was dissolved in 10 mL of ethanol, and 1.0 mL of this solution was added to the above mixture. This mixture was stirred for 6 h at 60 °C, after that, the prepared nanocatalyst was collected with a magnet, rinsed several times with EtOH, and dried at ambient temperature.

### General procedure for the hydrogeneration of nitroarenes

Under atmospheric pressure and room temperature, nitrobenzene compounds were hydrogenated in the presence of a catalytic system. In a 10 mL round-bottom flask, 1.0 mmol of nitrobenzene, 30 mg of MBC-PVIm/Pd nanocatalyst, and 5 mL of water as solvent were added. The above reaction mixture added 3 mmol of NaBH_4_ as the hydrogen donor. An external magnet was used to separate the nanocatalyst from the reaction mixture following the completion of the reaction. The reduction reaction was monitored using thin-layer chromatography (n-hexane: ethyl acetate 7:3).

Similarly, other aromatic nitro compounds were also hydrogenated using the prepared nanocatalyst under the same conditions.

### MBC-PVIm/Pd nanocatalyst Catalyzed Sonogashira Coupling

**Scheme 1:**
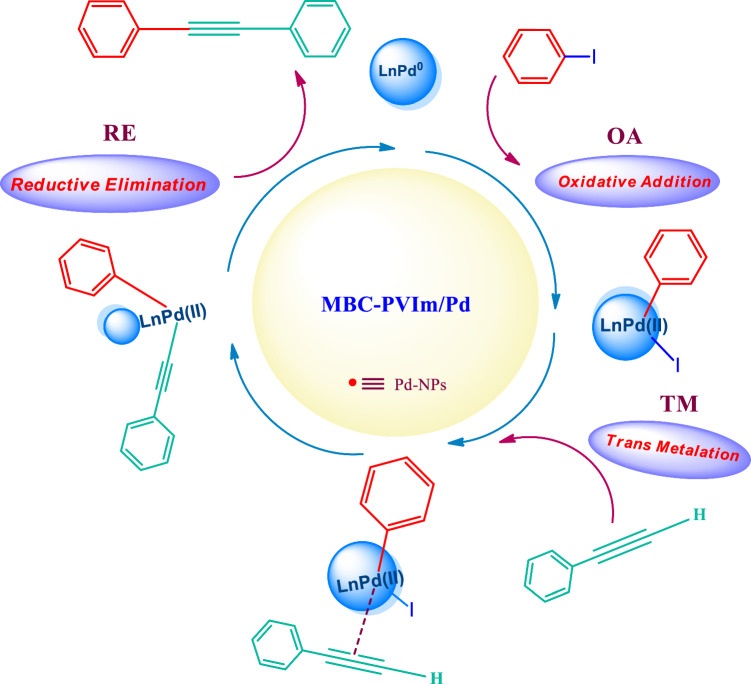
Passible mechanism for the Sonogashira coupling reaction using MBC-PVIm/Pd NPs.

To a suitable round-bottom flask, iodobenzene (1 mmol), phenylacetylene (1.2 mmol), and K_2_CO_3_ (2 mmol) were mixed with 3 ml water, along with 20 mg MBC-PVIm/Pd nanocatalyst. This mixture was stirred at 50 °C. With the use of TLC, the progress of the reaction was monitored. After the reaction completion, the nanocatalyst was separated by a magnet. Ethyl acetate was then used to extract the filtrate. Sodium sulfate was used to dry the organic part, then the solvent was removed by rotary evaporation under decreased pressure. The reaction mechanism was shown in Scheme 1.

## Result and discussion

### Characterization of MBC-PVIm/Pd nanocatalyst

**Figure 1:**
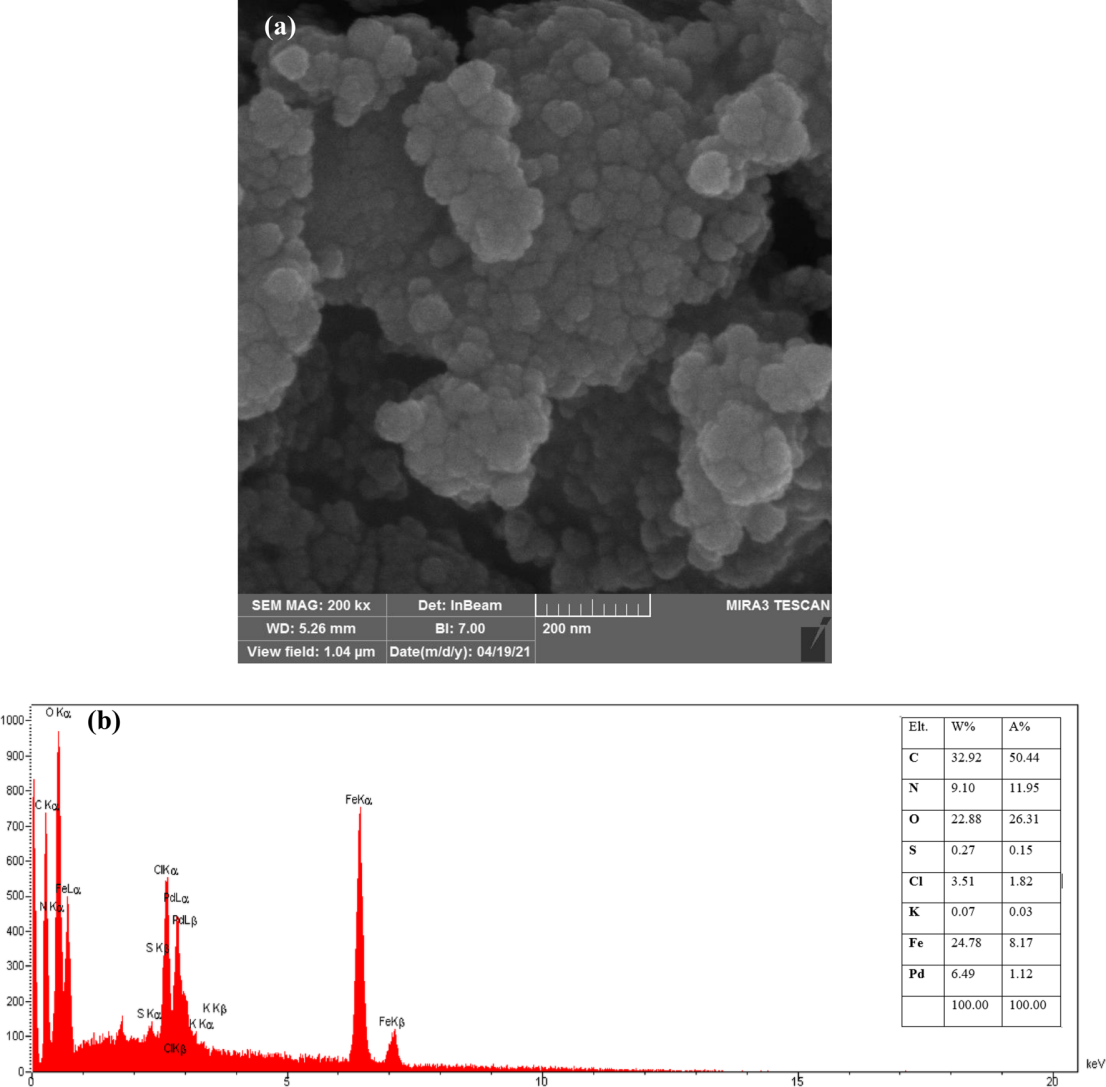
(**a**) FESEM, and (**b**) EDS of MBC-PVIm/Pd nanocatalyst.

In order to investigate the size of the synthesized nanocatalyst, a field emission scanning electron micrograph (FESEM) image was used. According to the FESEM image of the MBC-PVIm/Pd nanocatalyst, the Fe_3_O_4_, and Pd nanoparticles are almost finely dispersed on the all-round core, and in some zones were seen agglomerations of spherical particles. It was found that nanoparticles ranged in size from 25 to 50 nm. MBC-PVIm/Pd nanocatalyst was also analyzed by energy-dispersive X-ray (EDX) to affirm the presence of related elements. C, Pd, Fe, O, N, and S elements are present in the nanocatalyst. On the other hand, slight amounts of potassium and chlorine elements are present due to impurities in the biochar structure (Fig. 1).

**Figure 2:**
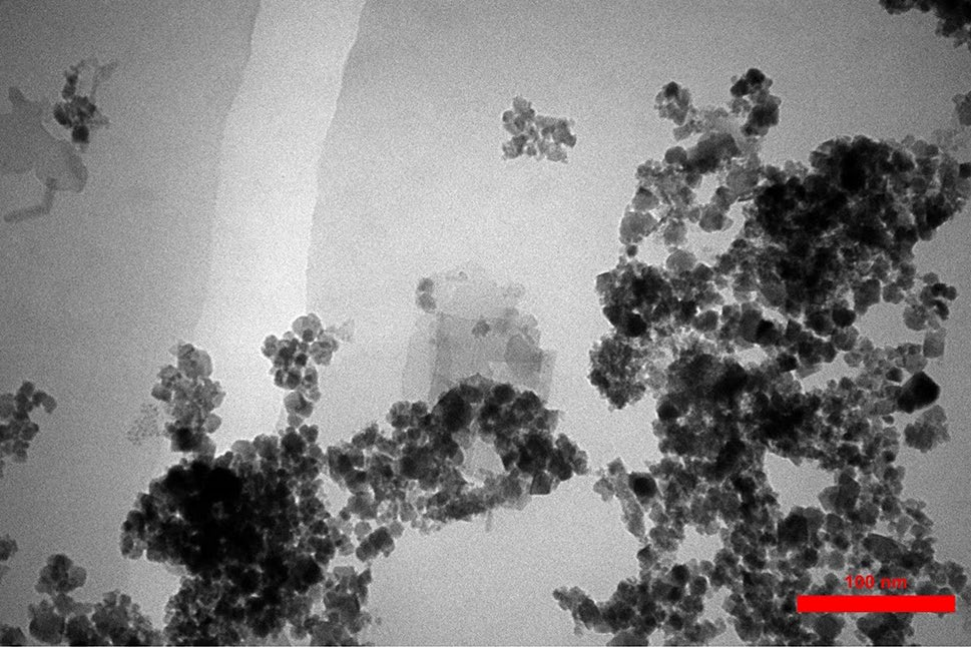
TEM image of MBC-PVIm/Pd nanocatalyst.

Figure 2 demonstrates TEM images obtained for more clarity and detail of the prepared nanocatalyst. Fe_3_O_4_ and Pd particles in this Fig. are nearly spherical and average 25 nm in diameter with a roughly spherical shape. These nanoparticles are almost uniformly dispersed on the biochar substrate.

**Figure 3:**
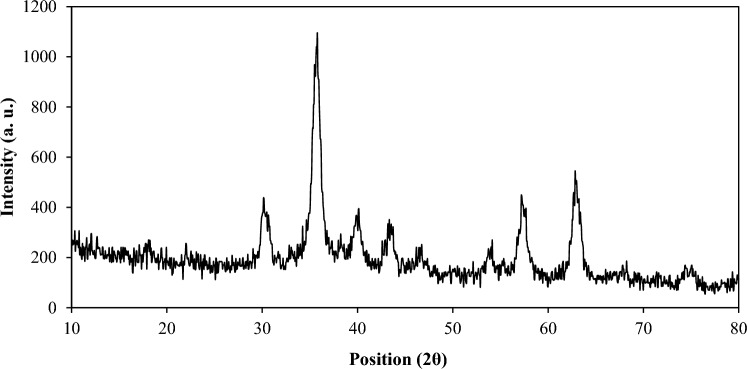
XRD pattern of MBC-PVIm/Pd nanocatalyst.

Figure 3 illustrates the results of the XRD pattern of the MBC-PVIm/Pd nanocatalyst. A weak peak at 22.04° with the plane (002) corresponds to biochar with a graphite-like structure. Peaks appeared at 2θ = 30.19°, 40.09°, 50.69°, 63.94°, and 74.94° related to the (220), (311), (400), (422), and (511) planes of Fe_3_O_4_ nanoparticles, respectively (JCPDS card no. 39-1346). The characteristic peaks of Pd nanoparticles at 40.09°, 46.59°, and 68.14°, correspond to the diffraction from the (111), (200), (220) planes (JCPDS No. 01-087-0639).

**Figure 4:**
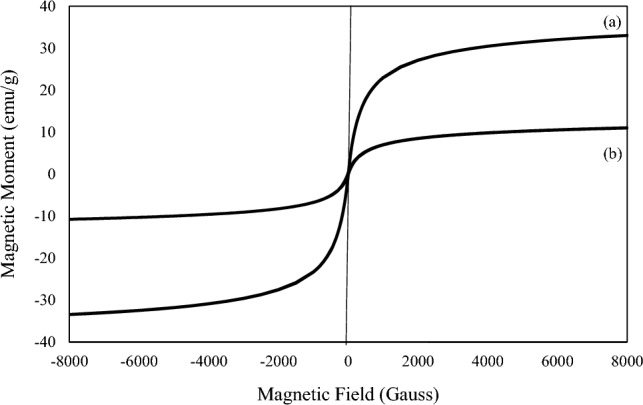
VSM of (**a**) Fe_3_O_4_, and (**b**) MBC-PVIm/Pd nanocatalyst.

At room temperature, a vibration sample magnetometer (VSM) was used to measure magnetization within a range of − 8000 to + 8000 Oe. Saturation magnetization values of 33 and 11 emu/g were observed in the magnetic hysteresis curve of Fe_3_O_4_ and the MBC-PVIm/Pd nanocatalyst respectively (Fig. 4). There was a smaller amount of magnetic saturation in MBC-PVIm/Pd nanocatalysts in comparison to Fe_3_O_4_, which contained organic and nonmagnetic components in this structure. Regardless of the decrease in Ms value, it could still be efficiently separated from the reaction mixture with a magnet.

**Figure 5:**
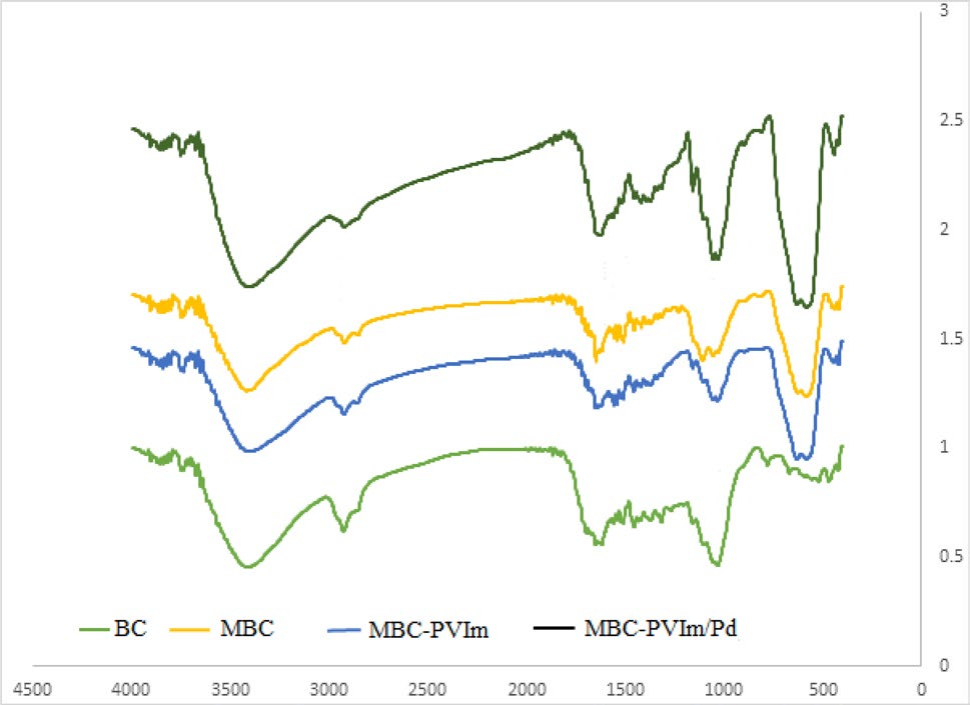
FTIR of various steps of nanocomposite synthesis.

The presence of functional groups in the compounds BC, MBC, MBC-PVIm, and MBC-PVIm/Pd was confirmed using FT-IR spectra (Fig. 5). The spectra of MBC show roughly 3388 cm^−1^ (–OH), 2927 cm^−1^ (–CH), and 1703 cm^−1^ (–C=O) characteristic groups, which is in excellent accord with the literature. The 1627 cm^−1^ (–C=C) and 1156 cm^−1^ (–CO) in BC are thought to have different structural purposes. Additionally, the Fe–O stretch vibration may be related to the MBC peak observed between 450 and 560 cm^−1^. The FTIR spectrophotometer cannot confirm the formation of MBC-PVIm/Pd even though the typical peaks of PVIm and Pd NP can be seen in the cast's FTIR spectra. This is because the spectra of these components overlap with the functional groups of biochar and Fe_3_O_4_.

### Optimization conditions of the hydrogenation reaction

Using nitrobenzene (1.0 mmol) as a model reaction, the reduction conditions of nitroaromatic compounds were optimized. Based on Table 1 we evaluated the amount of MBC-PVIm/Pd nanocatalyst, the solvent type, and the temperature. Hence, nanocatalysts were discussed in different amounts at the outset.

**Table 1:** Optimization conditions of the hydrogenation reaction.

Entry	Nanocatalyst (mg)	Temperature (°C)	Solvent	Time (min)	Yield (%)
1	–	25	H2O	30	–
2	5	25	H_2_O	30	20
3	10	25	H_2_O	30	40
4	25	25	H_2_O	30	65
5	10	50	H_2_O	30	55
6	20	50	H_2_O	30	70
7	30	50	H_2_O	30	99
8	40	50	H_2_O	30	90
9	30	70	H_2_O	30	90
10	20	60	EtOH	30	10
11	30	60	EtOH	30	20

Reaction conditions: Nitrobenzene (1.0 mmol), Solvent (5.0 mL), NaBH_4_ (3.0 mmol).

According to the results, a reduction reaction was not observed without the catalyst, demonstrating that MBC-PVIm/Pd nanocatalyst is required (Table 1, Entry 1). Therefore, 30 mg of MBC-PVIm/Pd was chosen as the optimum amount of nanocatalyst.

The effect of temperature on the reduction reaction was also investigated. This reaction was performed at 25, 50, 60, and 70 °C. The appropriate reaction temperature was chosen to be 50°C due to the higher reaction efficiency.

on the other hand, the model reaction was conducted with two solvents i.e., water and ethanol. Water showed the best results with a 99% yield, also due to its environmental friendliness and low cost, it was chosen as the optimal solvent.

In the next step, the entirety of the provided protocol was examined. For this purpose, nitroarenes with different electronic properties and 1-nitro naphthalene were used as substrates. In addition, to characterize the selectivity of the catalyst, the hydrogenation of 4-nitro acetophenone was also carried out, which reduced not only the nitro group but also the ketone group. The results in Table 2 showed that the used nitroarenes could perform the hydrogenation reaction to produce the corresponding product with high efficiency. However, the reaction yield was lower than that of nitrobenzene. Even this observation can be attributed to the 1-nitro naphthalene with a larger size, which does not allow effectively encloses and transfers to the aqueous environment. A comparison of the efficiency of hydrogenation of nitrobenzenes with other derivatives showed that substitution in the ortho position decreased the reaction efficiency.

**Table 2:** The reduction of nitroaromatic compounds in the presence of MBC-PVIm/Pd.

Entry	Nitroarene	Product	Time (min)	Yield (%)
1			30	99
2			70	80
3			55	85
4			70	93
5			120	55

Reaction conditions: Nitroarene (1.0 mmol), MBC-PVIm/Pd catalyst (30 mg), Water (5 mL), 50 °C, NaBH_4_ (3.0 mmol).

### Investigating the effect of nanocatalyst recyclability in the hydrogenation reaction

**Figure 6:**
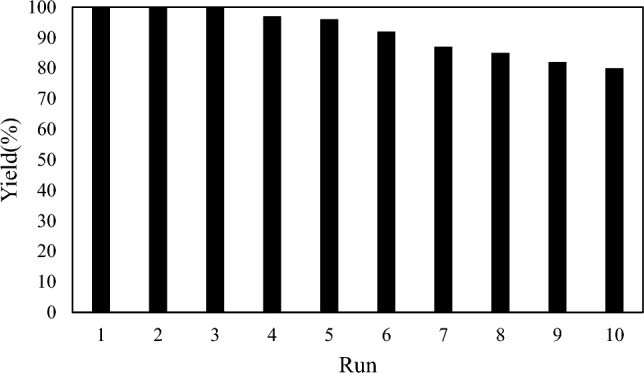
Recycling of nanocatalyst in the hydrogenation reaction.

To study the recyclability of the catalyst, at the end of the reaction, the nanocatalyst was collected magnetically, after washing and drying, the catalyst was used for the following consecutive times under the same reaction conditions. This recovery cycle of reuse of the catalyst was carried out for 10 consecutive reaction times and the performance of each run was measured and compared to the fresh catalyst as shown in Fig. 6. The results of catalyst recycling were excellent and only a small amount of loss of catalytic activity was observed after ten times of recycling. Satisfactorily, the ICP analysis of the recovered catalyst after ten consecutive reaction times showed that the washing has lost to a minor amount of MBC-PVIm/Pd, i.e., about 0.09% of the initial Pd loading. These observations confirmed the efficiency of the catalytic system designed for the MBC-PVIm/Pd.

### Optimization conditions of Sonogashira reaction

Initial experiments were conducted using MBC-PVIm/Pd nanocatalyst for the Sonogashira reaction of iodobenzene with phenylacetylene as a model reaction. The reaction was investigated using different conditions, including nanocatalyst amount, temperature, and type of solvent and base. The effect of solvents such as water and ethanol were studied in the model Sonogashira reaction (Table 3). Further study was conducted with water, a green and environmentally friendly solvent, which provided a high yield for producing the desired product.

**Table 3:** Optimization conditions of Sonogashira reaction.

Entry	Nanocatalyst (mg)	Temperature (°C)	Solvent	base	Time (min)	Yield (%)
1	10	50	H_2_O	K_2_CO_3_	15	70
2	20	50	H_2_O	K_2_CO_3_	15	95
3	30	50	H_2_O	K_2_CO_3_	15	95
4	20	25	H_2_O	K_2_CO_3_	15	35
5	10	100	H_2_O	K_2_CO_3_	15	80
6	20	50	EtOH	K_2_CO_3_	15	83
7	20	50	H_2_O	Na_2_CO_3_	15	15
8	20	50	H_2_O	Cs_2_CO_3_	15	85
9	20	50	H_2_O	NaOH	15	70
10	20	50	H_2_O	KOH	15	25

Reaction conditions. Aryl halide (1 mmol), Terminal alkyne (1.2 mmol), base (2 mmol), solvent (3 mL).

The nanocatalyst amount was evaluated and it was found that 20 mg of MBC-PVIm/Pd nanocatalyst enhanced the yield as compared to 10 mg, and 30 mg (Table 3). The yield did not change when the nanocatalyst amount was increased.

In order to understand the significance of bases in coupling reactions, a variety of bases were studied. K_2_CO_3_, Na_2_CO_3_, Cs_2_CO_3_, NaOH, and KOH were selected for the model reaction. A good yield was obtained with K_2_CO_3_ and used for further reactions (Table 3).

The effect of temperature on the reaction of the model was carried out. Temperatures of 25, 50, and 100 °C were investigated. After reaching 50 °C, the reactants were completely consumed for 15 min, resulting in excellent yields.

The optimum conditions were as follows: iodobenzene (1.0 mmol), phenylacetylene (1.2 mmol), 20 mg MBC-PVIm/Pd, K_2_CO_3_ (2 mmol), H_2_O (3 mL) at 50 °C for 15 min.

To investigate the catalytic activity, the MBC-PVIm/Pd nanocatalyst was applied in reactions with a variety of aryl halides and terminal alkenes, then the results were summarized in Table 4. Originally, different electron-donating and electron-withdrawing groups on aryl halides like methyl, methoxy, nitro, and ketone were investigated. The presence of electron-withdrawing and electron-donating groups on aryl halides led to the production of desired products with acceptable to excellent yields under optimal conditions. More results can be seen in Table 4.

**Table 4:**
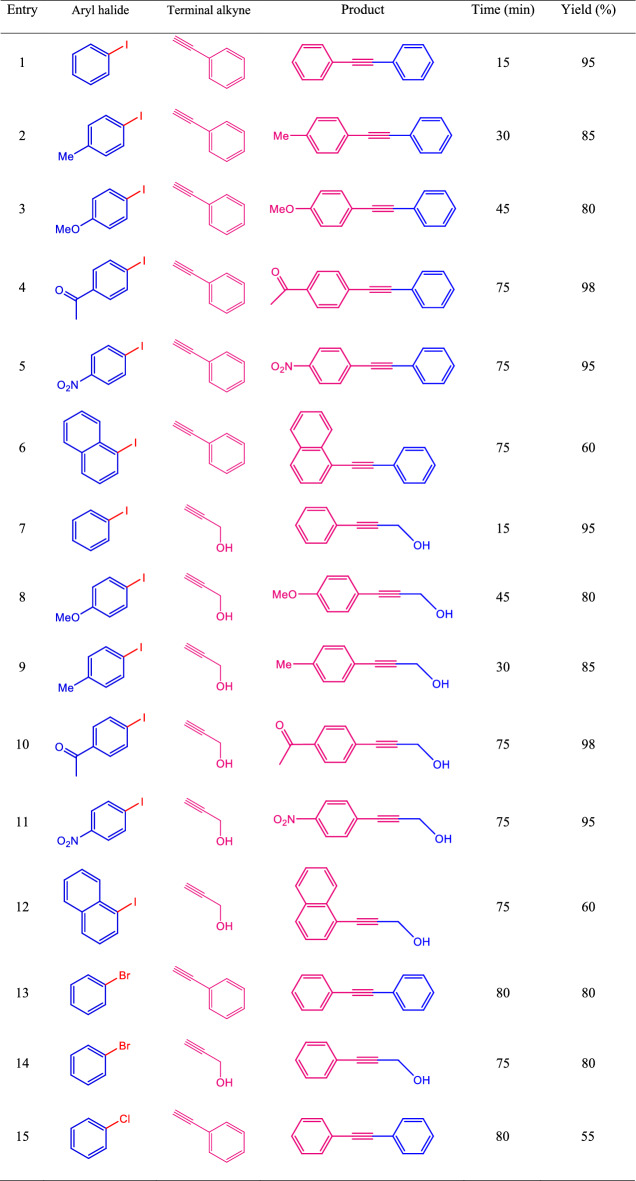
MBC-PVIm/Pd catalyzed Sonogashira cross-coupling reaction of aryl halides with terminal alkenes.

Reaction conditions: Aryl halide (1 mmol), Terminal alkyne (1.2 mmol), nanocatalyst (20 mg), water (3 mL) at 50 °C.

### Recycling procedure of the MBC-PVIm/Pd nanocatalyst for Sonogashira reaction

**Figure 7:**
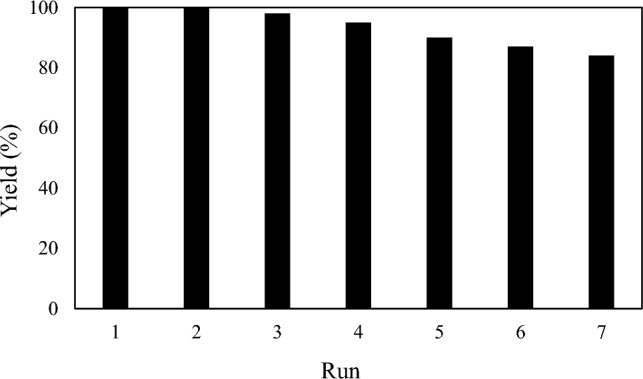
Catalyst recycling diagram in Sonogashira coupling reaction.

After the reaction, an external magnet was used to separate the MBC-PVIm/Pd nanocatalyst. A fresh substrate was then used directly under similar conditions without further purification after the catalyst was washed with ethanol, and dried. With little loss of catalytic activity, the recovered catalyst can be used for seven cycles. Figure 7 illustrates the plot of yield vs runs for seven cycles. MBC-PVIm/Pd presented in this study is stable.

The nitroarene hydrogenation and Sonogashira reactions were compared with some of those mentioned in the papers (Table 5) in order to determine the effectiveness of the MBC-PVIm/Pd nanocatalyst. According to the information in this Table, the reactions catalyzed by MBC-PVIm/Pd nanocatalyst produces a comparable or better yield and goes more quickly than other protocols.

**Table 5:** Comparison of present method with other reported processes.

	Entry	Catalyst	Yield%	Time (min)	References
Hydrogenation reaction	1	Co_1_/NPC	99	210	^38^
	2	rGO/MnFe2O4	99	30	^39^
	3	AO@PdNPs	100	120	^40^
	4	nZVI	90	–	^41^
	5	MBC-PVIm/Pd	99	30	This work
Sonogashira reaction	1	Pd	70	480	^42^
	2	Pd	80	240	^43^
	3	Pd/Cu	100	150	^43^
	4	Pd/C	90	120	^44^
	5	MBC-PVIm/Pd	95	15	This work

## Conclusions

In this study, magnetically separable and well-dispersed Pd nanoparticles modified biochar nanocatalyst was successfully prepared. Fe_3_O_4_ and Pd obtained on the surface of biochar improve the utilization of Pd as well as their separation from the solution by simply using an external magnet. MBC-PVIm/Pd nanocatalyst displayed excellent catalytic activity in the transfer hydrogenation of nitroarenes. In addition, this nanocatalyst was used to catalyze the Sonogashira reaction and the results showed the high efficiency of this nanocatalyst. The polymer-decorated biochar here can supply a high surface area to adsorb the reactants and substrate and increase the availability of the active sites Pd in the catalytic reaction process. The use of the aqueous solution as a solvent and NaBH_4_ as a reducing agent represents the catalytic system of the concept of sustainable green chemistry. Therefore, the work reported here provides an effective strategy to prepare magnetic catalysts modified with palladium and poly imidazole nanoparticles on biochar surfaces to catalyze the hydrogenation reactions of nitroarenes and sonogashira.

## Data Availability

All data generated or analyzed during this study are included in this published article.

## References

[1] Heaney F (2001). The nitro group in organic synthesis. Synthesis.

[2] Rao H, Fu H, Jiang Y, Zhao Y (2009). Easy copper-catalyzed synthesis of primary aromatic amines by couplings aromatic boronic acids with aqueous ammonia at room temperature. Angew. Chem..

[3] Yu J, Zhang P, Wu J, Shang Z (2013). Metal-free C-N bond-forming reaction: Straightforward synthesis of anilines, through cleavage of aryl C–O bond and amide C–N bond. Tetrahedron Lett..

[4] Klinkenberg JL, Hartwig JF (2011). Catalytic organometallic reactions of ammonia. Angew. Chem. Int. Ed..

[5] Tuteja J, Nishimura S, Ebitani K (2014). Base-free chemoselective transfer hydrogenation of nitroarenes to anilines with formic acid as hydrogen source by a reusable heterogeneous Pd/ZrP catalyst. RSC Adv..

[6] Morse JR, Callejas JF, Darling AJ, Schaak RE (2017). Bulk iron pyrite as a catalyst for the selective hydrogenation of nitroarenes. Chem Commun..

[7] Sanjini NS, Velmathi S (2014). Iron impregnated SBA-15, a mild and efficient catalyst for the catalytic hydride transfer reduction of aromatic nitro compounds. Rsc Adv..

[8] Yang LM, Huang LF, Luh TY (2004). Kumada− Corriu reactions of Alkyl halides with alkynyl nucleophiles. Org. Lett..

[9] Frigoli S, Fuganti C, Malpezzi L, Serra S (2005). A practical and efficient process for the preparation of tazarotene. Org. Process Res. Dev..

[10] Alberico D, Scott ME, Lautens M (2007). Aryl− aryl bond formation by transition-metal-catalyzed direct arylation. Chem. Rev..

[11] Li JH, Li JL, Wang DP, Pi SF, Xie YX, Zhang MB, Hu XC (2007). CuI-catalyzed Suzuki− Miyaura and Sonogashira cross-coupling reactions using DABCO as ligand. J. Org. Chem..

[12] Doucet H, Hierso JC (2007). Palladium-based catalytic systems for the synthesis of conjugated enynes by Sonogashira reactions and related alkynylations. Angew. Chem. Int. Ed..

[13] Lin L, Liu H, Zhang X (2017). ZnO-template synthesis of rattle-type catalysts with supported Pd nanoparticles encapsulated in hollow ZIF-8 for liquid hydrogenation. Chem. Eng. J..

[14] Verho O, Gustafson KP, Nagendiran A, Tai CW, Bäckvall JE (2014). Mild and selective hydrogenation of nitro compounds using palladium nanoparticles supported on amino-functionalized mesocellular foam. ChemCatChem.

[15] van Spronsen MA, Frenken JW, Groot IM (2017). Surface science under reaction conditions: CO oxidation on Pt and Pd model catalysts. Chem. Soc. Rev..

[16] Huang H, Wang X (2013). Design and synthesis of Pd–MnO_2_ nanolamella–graphene composite as a high-performance multifunctional electrocatalyst towards formic acid and methanol oxidation. Phys. Chem. Chem. Phys..

[17] Metin, Ö. *et al.* Ni/Pd core/shell nanoparticles supported on graphene as a highly active and reusable catalyst for Suzuki-Miyaura cross-coupling reaction. *Nano Res.* **6**, 10–18 (2013).

[18] Shi, Y. *et al.* 3D lithiophilic framework fixed on the surface of LLZTO solid electrolyte Shaping the Contact between Li Metal and Ceramic. *Chem. Eng. J.* **469**, 144090 (2011).

[19] Corma A, Garcia H (2008). Crossing the borders between homogeneous and heterogeneous catalysis: Developing recoverable and reusable catalytic systems. Top. Catal..

[20] Moradi P, Hajjami M, Tahmasbi B (2020). Fabricated copper catalyst on biochar nanoparticles for the synthesis of tetrazoles as antimicrobial agents. Polyhedron.

[21] Roberts KG, Gloy BA, Joseph S, Scott NR, Lehmann J (2010). Life cycle assessment of biochar systems: Estimating the energetic, economic, and climate change potential. Environ. Sci. Technol..

[22] Brown TR, Wright MM, Brown RC (2011). Estimating profitability of two biochar production scenarios: Slow pyrolysis vs fast pyrolysis. Biofuels Bioprod. Biorefin..

[23] Laird DA, Brown RC, Amonette JE, Lehmann J (2009). Review of the pyrolysis platform for coproducing bio-oil and biochar. Biofuels Bioprod. Biorefin..

[24] Titirici MM, White RJ, Falco C, Sevilla M (2012). Black perspectives for a green future: Hydrothermal carbons for environment protection and energy storage. Energy Environ. Sci..

[25] Datta S, Christena LR, Rajaram YR (2013). Enzyme immobilization: An overview on techniques and support materials. 3 Biotech.

[26] Ren X, Tang L, Wang J, Almatrafi E, Feng H, Tang X, Yu J, Yang Y, Li X, Zhou C, Zeng Z (2021). Highly efficient catalytic hydrogenation of nitrophenols by sewage sludge derived biochar. Water Res..

[27] Gu H, Gao Y, Xiong M, Zhang D, Chen W, Xu Z (2021). Removal of nitrobenzene from aqueous solution by graphene/biochar supported nanoscale zero-valent-iron: Reduction enhancement behavior and mechanism. Sep. Purif. Technol..

[28] Wei G, Zhang J, Luo J, Xue H, Huang D, Cheng Z, Jiang X (2019). Nanoscale zero-valent iron supported on biochar for the highly efficient removal of nitrobenzene. Front. Environ. Sci. Eng..

[29] Ahmad S, Gao F, Lyu H, Ma J, Zhao B, Xu S, Ri C, Tang J (2022). Temperature-dependent carbothermally reduced iron and nitrogen doped biochar composites for removal of hexavalent chromium and nitrobenzene. Chem. Eng. J..

[30] Ferlin F, Valentini F, Sciosci D, Calamante M, Petricci E, Vaccaro L (2021). Biomass waste-derived Pd–PiNe catalyst for the continuous-flow copper-free sonogashira reaction in a CPME–water azeotropic mixture. ACS Sustain. Chem. Eng..

[31] Kempasiddaiah M, Raj KS, Kandathil V, Dateer RB, Sasidhar BS, Yelamaggad CV, Rout CS, Patil SA (2021). Waste biomass-derived carbon-supported palladium-based catalyst for cross-coupling reactions and energy storage applications. Appl. Surf. Sci..

[32] Altavilla C, Sarno M, Ciambelli P (2009). Synthesis of ordered layers of monodisperse CoFe_2_O_4_ nanoparticles for catalyzed growth of carbon nanotubes on silicon substrate. Chem. Mater..

[33] Zeng T, Chen WW, Cirtiu CM, Moores A, Song G, Li CJ (2010). Fe_3_O_4_ nanoparticles: A robust and magnetically recoverable catalyst for three-component coupling of aldehyde, alkyne and amine. Green Chem..

[34] Karami B, Hoseini SJ, Eskandari K, Ghasemi A, Nasrabadi H (2012). Synthesis of xanthene derivatives by employing Fe_3_O_4_ nanoparticles as an effective and magnetically recoverable catalyst in water. Catal. Sci. Technol..

[35] Yang X, Chen W, Huang J, Zhou Y, Zhu Y, Li C (2015). Rapid degradation of methylene blue in a novel heterogeneous Fe_3_O_4_@[email protected] 2-catalyzed photo-Fenton system. Sci. Rep..

[36] Kooti M, Nasiri E (2015). Phosphotungstic acid supported on silica-coated CoFe_2_O_4_ nanoparticles: An efficient and magnetically-recoverable catalyst for N-formylation of amines under solvent-free conditions. J. Mol. Catal. A Chem..

[37] Zhu Z, Li X, Zhao Q, Li H, Shen Y, Chen G (2010). Porous, “brick-like” NiFe_2_O_4_ nanocrystals loaded with Ag species towards effective degradation of toluene. Chem. Eng. J..

[38] Jin H, Li P, Cui P, Shi J, Zhou W, Yu X, Song W, Cao C (2022). Unprecedentedly high activity and selectivity for hydrogenation of nitroarenes with single atomic Co_1_-N_3_P_1_ sites. Nat. Commun..

[39] Fuladi V, Bezaatpour A, Azizian-Kalandaragh Y, Amiri M, Nuri A, Nouhi S, Taffa DH, Wark M (2022). Photocatalytic aspect of rGO/MnFe_2_O_4_ as an efficient magnetically retrievable catalyst for reduction of nitroaromatic compounds under visible-light irradiation. J. Environ. Chem. Eng..

[40] Abdullah FO, Behrouzi L, Kaboudin B (2021). A novel synthesis of highly stable palladium nanoparticles and their application in the reduction of nitroaromatic compounds. Mater. Res. Express.

[41] Shukla F, Kikani T, Khan A, Thakore S (2022). α-Hydroxy acids modified β-cyclodextrin capped iron nanocatalyst for rapid reduction of nitroaromatics: A sonochemical approach. Int. J. Biol. Macromol..

[42] Platonova YB, Volov AN, Tomilova LG (2019). Palladium (II) octaalkoxy-and octaphenoxyphthalocyanines: Synthesis and evaluation as catalysts in the Sonogashira reaction. J. Catal..

[43] Gazvoda M, Virant M, Pinter B, Košmrlj J (2018). Mechanism of copper-free Sonogashira reaction operates through palladium-palladium transmetallation. Nat. Commun..

[44] Heidenreich RG, Koehler K, Krauter JG, Pietsch J (2002). Pd/C as a highly active catalyst for Heck, Suzuki and Sonogashira reactions. Synlett.

